# Unveiling extracellular matrix assembly: Insights and approaches through bioorthogonal chemistry

**DOI:** 10.1016/j.mtbio.2023.100768

**Published:** 2023-08-07

**Authors:** Shima Tavakoli, Austin Evans, Oommen P. Oommen, Laura Creemers, Jharna Barman Nandi, Jöns Hilborn, Oommen P. Varghese

**Affiliations:** a Macromolecular Chemistry Division, Department of Chemistry–Ångström Laboratory, Uppsala University, 751 21, Uppsala, Sweden; b Bioengineering and Nanomedicine Group, Faculty of Medicine and Health Technologies, Tampere University, 33720, Tampere, Finland; c Department of Orthopedics, University Medical Center Utrecht, 3584, CX, Utrecht, the Netherlands; d Department of Chemistry, Sarojini Naidu College for Women, 30 Jessore Road, Kolkata, 700028, India

**Keywords:** Bioorthogonal chemistry, Extracellular matrix, Metabolic labeling, Visualizing cells

## Abstract

Visualizing cells, tissues, and their components specifically without interference with cellular functions, such as biochemical reactions, and cellular viability remains important for biomedical researchers worldwide. For an improved understanding of disease progression, tissue formation during development, and tissue regeneration, labeling extracellular matrix (ECM) components secreted by cells persists is required. Bioorthogonal chemistry approaches offer solutions to visualizing and labeling ECM constituents without interfering with other chemical or biological events. Although biorthogonal chemistry has been studied extensively for several applications, this review summarizes the recent advancements in using biorthogonal chemistry specifically for metabolic labeling and visualization of ECM proteins and glycosaminoglycans that are secreted by cells and living tissues. Challenges, limitations, and future directions surrounding biorthogonal chemistry involved in the labeling of ECM components are discussed. Finally, potential solutions for improvements to biorthogonal chemical approaches are suggested. This would provide theoretical guidance for labeling and visualization of de novo proteins and polysaccharides present in ECM that are cell-secreted for example during tissue remodeling or *in vitro* differentiation of stem cells.

## Graphical abstract

The visualization and labeling of ECM components secreted by cells are crucial for understanding tissue regeneration. Bioorthogonal chemistry approaches present a promising solution for labeling and visualizing ECM without interfering with other chemical or biological processes. This review focuses on recent advancements in using biorthogonal chemistry for metabolic labeling and visualization of ECM constituents.

**Figure:**
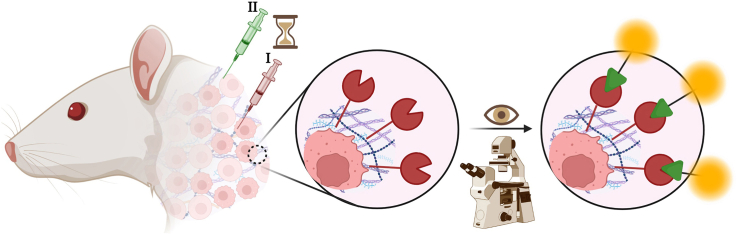

## 1 Introduction

**Fig. 1:**
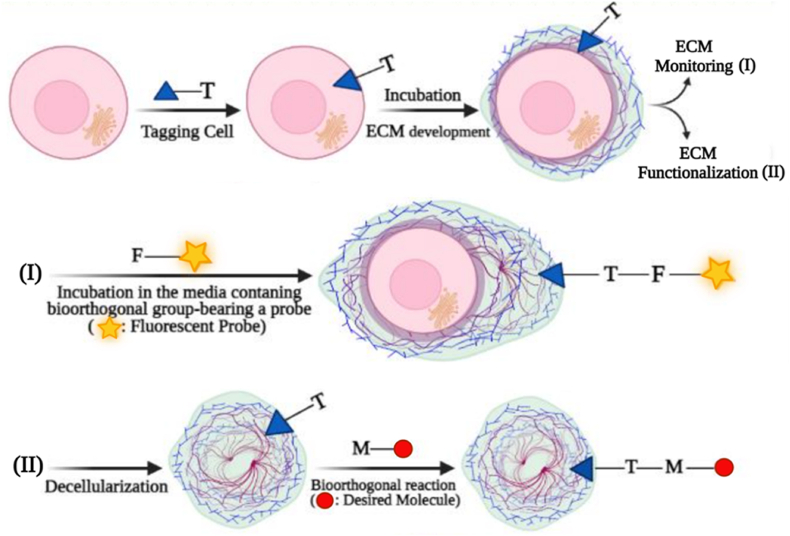
The schematic representation of labeling ECM for its visualization (I) or functionalization (II) with bioorthogonal functionalities. In the first step, the tagging blocks bearing a bioorthogonal functional group are incorporated into the target cell which leads to metabolic labeling of the cell surface ECM with the tag. For monitoring ECM production, the bioorthogonal functional group is reacted with a probe containing a complementary bioorthogonal group to visualize ECM status in real-time. For ECM functionalization, the matrix is decellularized, and then, functionalized with the desired molecule through bioorthogonal chemistry. The letters T, F and M represent the functional groups for click chemistry.

ECM is a fundamental noncellular structure present throughout the body, allowing for complex organization of all cell and tissue types, and is required for the biomechanical functions of the body [1]. ECM provides support and anchorage for the cell's morphology and it determines the cell's dynamic and function including cell viability, cell differentiation, cell polarity, cell proliferation, and cell migration. Moreover, the ECM provides physical and mechanical support for tissues, tissue integrity, and elasticity. It also has a critical role in growth, regenerative, and healing processes in tissue engineering and the regenerative medicine field [2,3]. The tissue engineering triangle includes three elements, reparative cells that can create a functional matrix, an appropriate scaffold for embedding cells and transplantation, and bioactive molecules, such as growth factors and cytokines to produce tissue-like grafts [4]. These components are commonly used for efficient regeneration by depositing ECM and repairing lost tissue. Therefore, quantitative and qualitative monitoring of ECM is critical for the understanding of the engineering and regeneration processes. To characterize the ECM formation and consequently to understand the tissue regeneration process, some methods such as histological and optical analysis are being used [5]. Although the classical methods of histology are applied in the majority of pre-clinical and clinical studies, they are invasive and have destructive endpoints. Such approaches also require a high number of samples for repetition and they do not allow *in situ* monitoring of ECM formation. Moreover, histological staining is not the best option for quantification as the molecular weight of the stained ECM polymer, its charge state, and the ionic content of the explanted sample can interfere with achievable color intensity, leading to an incorrect conclusion [6]. Recently, bioorthogonal chemistry has been used as a complementary method for labeling biomolecules produced by living cells, enabling *in situ* visualizations of ECM with a noninvasive approach. However, it requires site-specific metabolic labeling of the ECM component. This method also allows cell-derived matrix functionalization selectively, which can be used as a scaffold biomaterial or could be used to simply modify the cell surface with a target ligand [7]. Briefly, this strategy is a two-step process; first building blocks modified with a bioorthogonal functional group are incorporated in cell products such as proteins or glycans. This can lead to metabolic tagging of structures on the cell membrane, e.g. on the glycocalyx or the proteoglycans present in the ECM. Thereafter, a probe bearing a complementary bioorthogonal functional group is added which reacts with the metabolically incorporated molecules on/in the cell. This method also enables the functionalization of a cell-derived matrix, which necessitates decellularization before conjugation. The resulting functionalized matrix can then serve as a scaffold with enhanced properties for tissue engineering applications (Fig. 1). Thus, bioorthogonal chemistry can be applied for a wide range of bio-applications including developments of active scaffolds for tissue engineering approaches and monitoring ECM formation for the regenerative medicine purposes.

In this review, we focus on bioorthogonal chemistry to metabolically label cells and in particular, discuss cell metabolic labeling of glycans and proteins to monitor ECM formation or to functionalize ECM.

## 2 ECM structure and components

**Fig. 2:**
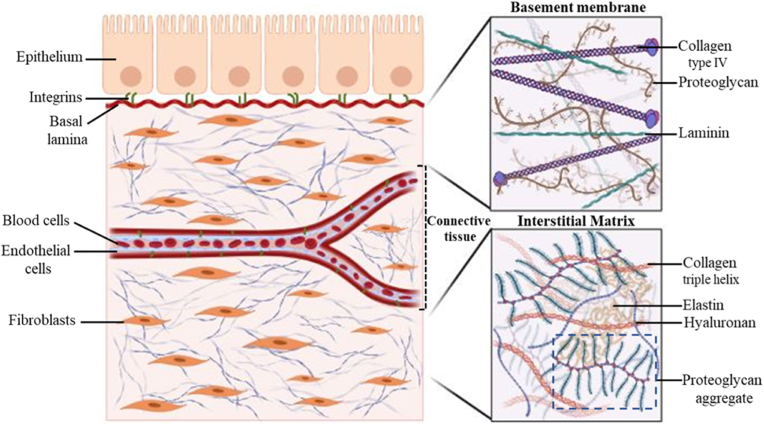
The schematic representation of the ECM structure. The ECM is subdivided into the pericellular matrix and interstitial matrix which each has its particular components. The basement membrane is mostly composed of type IV collagen, laminin, and proteoglycans and the interstitial matrix contains collagen triple helix, hyaluronan, elastin, and proteoglycan aggregates.

ECM is the primary component of connective tissues such as tendons, adipose tissue, bone, intervertebral disc (IVD), and cartilage [8]. The ECM is composed of proteins, including glycoproteins and proteoglycans, and carbohydrate polymers that support and surround the cells. ECM plays a significant role in several physiological and pathological processes, such as embryonic development, hemostasis, fibrosis of various organs, tumor growth and metastasis, inflammation, wound healing, and angiogenesis [9,10]. Every organ has its unique ECM composition and topology that is generated in the early embryonic stages [11,12] and then remodeled by cells in response to external stimuli and cell signaling molecules that are entrapped within ECM. Basement membranes are mainly composed of collagen type IV, laminin, and glycoproteins and are specialized sheet-like structures on which epithelial cells rest. In contrast, the interstitial ECM of connective tissues are mostly composed of collagen triple helix, elastin, hyaluronan and proteoglycan aggregate. In most connective tissues, the ECM constituents are produced by fibroblasts, but in several particular types of connective tissues, like cartilage and bone, the ECM is secreted by chondrocytes and osteoblasts [3]. In these connective tissues of the musculoskeletal system, ECM has a critical biochemical and biomechanical function, in addition to regulating diverse cellular processes [13]. Fundamentally, the interstitial ECM includes the interstitial matrix and the pericellular matrix which are a well-organized network of proteins and polysaccharides (Fig. 2).

### 2.1 Hyaluronan and proteoglycans

Hyaluronan or Hyaluronic acid (HA) is a linear non-sulfated glycosaminoglycan (GAG) containing repeating disaccharide units of d-glucuronic acid and N-acetyl-d-glucosamine [12]. HA is the only GAG that is biosynthesized at the cell membrane and not at the Golgi apparatus. HA is the main component of the pericellular matrix of many cell types and it can bind to its synthases as well as to its cellular receptors, influencing several cell functions. Moreover, HA also exists intracellularly in the nucleus of normal and tumor cells, although its exact function is not known [14,15]. Since HA can absorb a large amount of water, it provides tissues with remarkable plasticity, promotes cell regeneration, and stimulates the production of collagen [16]. Therefore, in tissue regeneration and development, extensive HA accumulation is observed.

Proteoglycans are composed of GAG chains that are covalently linked to a protein core, filling the majority of the extracellular interstitial space within the tissue in the form of a hydrated gel [17]. These ECM components contain binding motifs for growth factors, cell surface receptors such as integrins, and other ECM proteins. They have a variety of functions that reflect their unique buffering, hydration, protein binding, and force-resistance characteristics [18]. Proteoglycans are pivotal in maintaining fundamental cellular functionality. They fulfill critical roles such as promoting cell adhesion, facilitating cell migration, mediating signaling processes, and providing mechanical support. Moreover, proteoglycans play a key role in orchestrating the organization of the ECM and facilitating the formation of its supportive structure. Moreover, their ability to interact with growth factors, cytokines and chemokines, other ECM molecules, and cells, confers important signaling roles [12,19,20]. Proteoglycans have been placed into four categories including intracellular, cell-surface, pericellular, and extracellular proteoglycans [19]. Serglycin is the only intracellular proteoglycans. This unique proteoglycan forms a class on its own as it is the only proteoglycan that carries heparin side chains. In the cell-surface proteoglycan class there are thirteen genes, seven encoding transmembrane proteoglycans. Cell-surface proteoglycans interact with numerous regulators of cell behavior through their glycosaminoglycan chains [21]. Pericellular proteoglycans are closely associated with the surface of many cell types anchored via integrins or other receptors, but they can also be a part of most basement membranes. Extracellular proteoglycans are the largest family of proteoglycans in the tissues and the main members of this family are aggrecan, neurocan, versican, and brevican, which share common structural characteristics [12,22].

### 2.2 ECM proteins

Proteins are linear macromolecules that fold into a particular three-dimensional (3D) conformation as determined by the sequence of the amino acids in the polypeptide chain [23,24]. Collagen, elastin, fibronectin, and laminin are the main fibrous ECM proteins composed of particular amino acid sequences that are present in different isoforms [25]. Table 1 summarizes the role of major ECM proteins along with the main amino acids in their structures that may attributes to specificity of metabolic labeling.

**Table 1:** The ECM components and their main building blocks as well as the predominant role of each component.3.Cell-secreted ECM.

Type of components	Main role	Most abundant amino acids/GAGs	references
Proteins			
Collagen	• Cell adhesion and regulating cell function • Tissue development and regeneration	Glycine, Proline and Hydroxyproline	[25,27]
Elastin	• Structural integrity along with reversible extensibility and deformability • Support flexibility and repeated stretching	Glycine, Proline Valine, Alanine, Leucine, Desmosine and Isodesmosine	[[38], [39], [40]]
Fibronectin	• Cell adhesion, growth, migration, and differentiation • Blood clotting formation and wound healing	Arginine, Glycine, Aspartate, Leucine, and Glutamic acid	[30,41]
Fibrillin-1	• Provide sheath around elastin in the formation of elastic fibers	Cysteine, and Glycine	[12]
Laminin	• Provide cell-to-ECM interactions • Support embryonic development, angiogenesis, and organogenesis	Aspartate, Glycine, Arginine, Serine, Tyrosine, and Isoleucine	[33,42]
Tenascin	• Modulate inflammation and fibrosis	Cysteine and Arginine, Glycine, and Aspartate	[37,43]
Vitronectin	• Adhesive protein • Blood coagulation and wound healing	Arginine, Glycine, and Aspartate	[12,44]
Pericellular Proteoglycans			
Perlecan	• Essential for normal growth plate development and long bone growth • Essential for cartilage and cephalic development • Maintaining the endothelial barrier function	Heparan sulfate/Chondroitin sulfate	[45]
Agrin	• Promoting the aggregation of acetylcholine receptors	Heparan sulfate/Chondroitin sulfate	[46,47]
XVIII collagen	• Negative regulator of angiogenesis and an anti-atherosclerotic factor • Functioning as a key structural constituent required for the stabilization of skeletal muscle cells and microvessels	Heparan sulfate/Chondroitin sulfate	[19]
Extracellular Proteoglycans			
Aggrecan	• Major proteoglycan of cartilage • Forming massive macro-aggregate structures • protein-stabilized ternary structures with HA • Support resistance to compression of tissues	Chondroitin sulfate/Keratan sulfate	[48]
Versican	• Regulate cell adhesion, migration, and inflammation • Stabilize large supramolecular complex at the plasma membrane zone	Chondroitin sulfate/Dermatan sulfate	[49,50]
Neurocan	• Key ECM component of the central nervous system • Inhibiting neurite outgrowth *in vitro*	Chondroitin sulfate	[51,52]
Brevican	• One of the most important hyalectans of the central nervous system. • Maintain the extracellular environment of mature brain	Chondroitin sulfate	[53,54]
Biglycan	• Bind transforming growth factor beta (TGFβ) and modulates its bioactivity • Acting as a pro-angiogenic stimulus	Chondroitin sulfate	[55]
Decorin	• Regulate the bioactivities of cell growth factor and participates in ECM assembly • Act as a ligand of various cytokines and growth factors • Modulate vital roles in cancer cell proliferation, spread, pro-inflammatory processes and anti-fibrillogenesis	Dermatan sulfate	[56]
Epiphycan	• Epiphycan has a precise spatiotemporal distribution during cartilage development • Localizing to the entire growth plate, • Stimulate chondrogenesis	Dermatan sulfate/Chondroitin sulfate	[19]
Fibromodulin	• Bind to collagens I and II and delay fibril formation • Modulate the tumor stroma by increasing extracellular fluid volume and lowering interstitial fluid pressure • Promote angiogenesis	Keratan sulfate/Chondroitin sulfate	[57,58]
Lumican	• Regulate alignment and overall structure of collagen fibrils during murine tendon development • Inhibiting melanoma progression, and blocking melanoma cell adhesion • Inhibit MMP activity	Keratan sulfate	[59]
Keratocan	• Regulate proliferation and modulation of osteoprogenitor lineage differentiation	Keratan sulfate	[60]
Testican, 1-3	• Regulate extracellular protease cascades and neuronal function	Heparan sulfate/Chondroitin sulfate	[61,62]

Collagen, which is comprised of three polypeptide α chains, constitutes up to 30% of the total protein mass of a multicellular animal and is the most abundant fibrous protein in the interstitial matrix [9]. Tensile strength, cell adhesion regulation, chemotaxis, migration support, and direct tissue development and regeneration are the main role of collagen in ECM [18]. Mainly connective tissue cells synthesize and secrete collagen in the ECM. By exerting tension on the matrix, fibroblasts organize collagen fibrils into sheets which significantly influences the alignment of collagen fibers. The tertiary protein structural indicator of all collagens is their triple helix braid which is a tight right-handed helix of three α-chains and each contains one or more regions characterized by a repeating amino acid motif, Glycine-Xaa-Yaa [9]. This triple-helical organization is due to the repetitive patterning of Gly-Xaa-Yaa where Xaa and Yaa are predominantly proline and hydroxyproline, respectively (they constitute approximately 23% of the total of amino acids in collagen) [26]. There are 29 different collagen types formed by at least forty-six distinct polypeptide chains (α chains) in vertebrates. Besides, collagens can be categorized into seven categories according to their common domain homology and functions. These groups are fibrillar and network-forming collagens, fibril-associated collagens with interrupted triple helices, membrane-associated collagens with interrupted triple helices, anchoring fibrils, beaded-filament-forming collagens, and multiple triple-helix domains and interruptions/endostatin-producing collagens [27].

Elastin fibers are another essential component of ECM which is mostly composed of over 75% of glycine, valine, alanine, and proline and other amino acids depending on tissue source and developmental stage of elastin [28]. Elastin is a crosslinked polymer network of the monomeric-secreted precursor tropoelastin [29]. Tropoelastin is a 60–70 kDa protein that contains intermittent hydrophobic and lysine-containing crosslinking domains. Elastin provides recoil or elasticity to tissues that undergo repetitive tension and stretching such as blood vessels, skin, and elastic cartilage [12]. Elastin fibers in ECM are covered by glycoprotein microfibrils [18]. Microfibrils localize in the periphery of the fiber in adult tissues and are more complex in composition containing several proteins. The major constituents of microfibrils are cysteine-rich glycoproteins of large size, mainly fibrillin (fibrillin-1, -2, and, -3). Most fibrillins likely perform structural roles in microfibril assembly, while other associated proteins might perform regulatory functions rather than structural roles.

Other key proteins within the ECM structure include fibronectin and laminin which function as bridges between structural ECM molecules to reinforce its organization and to connect the soluble molecules to cells within the extracellular space [25]. Fibronectin is composed of two subunits that are covalently connected at their C-termini by a pair of disulfide bonds. These two subunits consist of three different types of modules, type I, II, and, III repeats. Fibronectin contains 12 type I repeats, two type II repeats, and 15–17 type III repeats, which together account for approximately 90% of the fibronectin sequence. All types of fibronectin repeating units are found in other molecules, suggesting that fibronectin evolved through exon shuffling [30]. In terms of solubility, fibronectin can be divided into two groups, soluble plasma fibronectin, and cellular fibronectin molecules. Cellular fibronectin molecules are a far more heterogeneous population due to cell type-specific and species-specific splicing [30,31]. The functional form of fibronectin *in vivo* is a fibrillar state that produces an interconnected network. Cells can assemble soluble fibronectin derived from fibronectin in plasma into fibers and cells can also release their fibronectin, which is secreted and shaped into fibers [32]. A variety of stimuli can produce or up-regulate the fibronectin matrix fibers. To maintain the presence of pre-existing fibronectin matrix, continuous and dynamic fibronectin matrix production is needed. Fibronectin matrix is produced at times of dynamic tissue remodeling, formation, or repair and is essential during embryonic development. The fibronectin matrix is also intensely up-regulated around tumor vasculature and appears to contribute to tumor progression [12].

Laminin, the other structural component of ECM is a large non-collagenous heterotrimeric protein. Each laminin heterotrimer consists of one α, one β, and one γ chain. They are assembled in basement membranes. The α-, β-, and γ-chains share structural homologies, including the laminin N-terminal domains (LN), laminin 4 domains (L4), laminin epidermal growth factor (EGF)-like repeats domains (LE), and the α-helical coiled-coil long arms. Laminin molecules interact with each other, and with other ECM components, and resident cells participate in the organization of ECMs and cell adhesion [12,33]. For example, laminins bind to each other through laminin LN domains promoting self-assembly into higher-order networks of polymers found in basement membranes. Laminin has a critical role in early embryonic development and organogenesis [33]. The distribution of laminin isoforms is tissue-specific. This indicates that laminins provide specific functions in different tissues [34]. They influence cell differentiation, adhesion, and migration, and are important for the maintenance and survival of tissues. For instance, laminins are upregulated in the wounded epithelium. The unique 3D structure of the protein provides the substrate for the epithelial cells to adhere to and move to cover the injured area to reestablish the intact epithelium. They also play an important role in angiogenesis as they are vital components of endothelial basement membranes contributing to vascularization [34,35].

Tenascins are another family (Tenascin-C, Tenascin-R, Tenascin-W, Tenascin-X, and Tenascin-Y) of proteins that can be found in ECM. In contrast to many other ECM proteins, tenascin displays highly restricted and dynamic patterns of expression in the embryo, particularly during neural development, vascularization, and skeletogenesis [36]. Tenascin proteins, depending on their mode of presentation (i.e., soluble or substrate-bound) and the cell types and differentiation states of the target tissues, convey different cellular functions. Tenascin molecules are re-expressed in the adult during normal processes such as nerve regeneration, wound healing, and tissue involution, and also in pathological states including metastasis, vascular disease, and tumorigenesis [37].

Biomaterials encapsulated with stem cells have been extensively utilized for different tissue engineering applications. However, it is important to recognize that these cells actively contribute to the formation of their matrix over time [63]. This cell-secreted matrix plays a significant role in shaping the microenvironment at the cell-biomaterial interface. It serves as a crucial component, especially when the biomaterial acts as a temporary scaffold during the development of new tissue and provide valuable insight on the differentiation pattern of the surrounding stem cells [64]. To fully harness and understand this process, it is imperative to evaluate the spatial and temporal deposition of the ECM and comprehend its composition. This knowledge can provide valuable insights into guiding cell behavior at different time points.

Existing assays for visualizing and quantifying ECM dynamics often fall short in terms of providing the required spatiotemporal resolution. Common techniques such as biochemical assays and immunofluorescence have limitations in this regard. Similarly, assays utilizing radiolabeled isotopes, such as autoradiography, can be complex and challenging. Furthermore, these conventional methods typically offer limited insights into the structure and organization of the secreted ECM [65]. In recent advancements, bioorthogonal chemistry has emerged as a promising approach for labeling cell-secreted ECM and tissue development. The bioorthogonal-based strategies allow researchers to label specific ECM proteins and polysaccharides, providing a comprehensive view of the ECM composition.

In the subsequent sections, we delve into an in-depth exploration of using bioorthogonal chemistry to label and study ECM components, shedding light on its overall potential and applications.

## 3 Bioorthogonal chemistry for metabolic labeling of cell-secreted ECM

A great effort has been made to label and probe biomolecules such as nucleic acids, lipids, proteins, and glycans in living systems. Genetically encoded peptide tags have expanded the repertoire of proteins that can be tagged in living systems. Other biomolecules including glycans, lipids, nucleic acids, and various molecules are, however, not amenable to such genetically encoded tags [66]. To address this challenge, bioorthogonal chemical labeling methods were developed that provided a complimentary and powerful tool for labeling and monitoring biomolecules in living cells and whole tissue/organisms without genetic manipulation [7]. Bioorthogonal chemistry is a class of chemical reactions that proceed rapidly. under the physiological environment but do not interfere with biological molecules or processes [67]. The term ‘bioorthogonal chemistry’ was coined by Bertozzi et al. who was recently awarded the Nobel Prize in Chemistry because of its widespread application in chemistry and biology. One of the advantages of these reactions is that it allows metabolic labeling of specific cell-surface or ECM-derived glycosaminoglycans which can be tagged by bioorthogonal chemical reporters. Such a strategy allows functional readout of cellular processes or for performing chemical reactions *in vivo* without inducing any toxicity [[68], [69], [70]]. A key highlight for these reactions is the design of biomolecules that allows site-specific cellular labeling of molecules. For instance, the metabolite can be a nucleoside for DNA labeling, a monosaccharide for glycan labeling, an amino acid for protein labeling, or a fatty acid for lipid labeling [66]. In a general strategy for labeling biomolecules using bioorthogonal chemistry, one of the two bioorthogonal functional moieties is incorporated onto the monomeric building block of the biomolecule (e.g., monosaccharides or amino acids) and delivered to the target cells. The biorthogonal group is then metabolically incorporated into the target biomolecule and is usually expressed on the cell membrane. Then, in the second step, the incorporated bioorthogonal groups (chemical tags) covalently interact with a probe bearing the complementary bioorthogonal functionality [70]. In this approach, the synthetic building blocks are incorporated into the living environment and tag all the biomolecules containing that building block. This approach is easy to use with a high-efficiency rate and with broad applicability. Therefore, it is an appropriate method for monitoring the dynamic changes in tissue remodeling, enabling online monitoring of ECM formation in tissue engineering and regenerative medicine applications [7]. The most popular bioorthogonal reactions for labeling ECM metabolites are azide and alkyne reactions, which were discovered by Barry Sharpless the co-recipient of the Nobel Prize in Chemistry 2022 who coined the term ‘click chemistry’ due to its high efficiency, simplicity, and selectivity Such reactions can undergo copper(I)-catalyzed azide-alkyne cycloaddition (CuAAC) that takes advantage of the formation of a copper acetylide to activate terminal alkynes toward reaction with azides. In the terms of efficiency, simplicity, and selectivity, the CuAAC reaction has all the features of “click chemistry” [71]. This cycloaddition between azides and alkynes can also be catalyzed by ruthenium (II) [72]. Although the copper(I)-catalyzed cycloaddition reaction rate is roughly seven orders faster than the uncatalyzed cycloaddition, the use of CuAAC in living systems has been hindered by the toxicity of copper(I) [73]. Therefore, the development of azide and alkyne coupling reactions continues and a series of cyclooctynes allowing detection of azides in living systems through the strain-promoted [3 + 2] cycloaddition without any catalyzer was developed [66]. However, the first-generation strain-promoted cycloaddition reactions had a considerably slower rate than CuAAC. To modify the kinetic of copper-free azides-alkynes reactions a series of compounds containing electron-withdrawing fluorine atoms at the propargylic positions were evaluated. Results demonstrated that the addition of difluorinated cyclooctyne (DIFO) increased the reaction rate as its kinetic was comparable to CuAAC. This prompted the emergence of “copper-free click chemistry” and DIFO–fluorophore conjugates enabled imaging of azide-labeled biomolecules within complex biological systems, including live cells [74]. Besides, more water-soluble azacyclooctyne was synthesized to improve other attributes of the cyclooctyne reagents such as pharmacokinetic properties [75]. For instance, dibenzocyclooctynein (DBCO) copper-free click reactions have a similar kinetic to DIFO while it is not toxic for cells [66]. Although catalyst-free cycloaddition reactions are very promising, the reaction is not always very selective. The strained octyne can also reacts with other reactive nucleophiles present in the biological milieu such as thiols to yield thiol-yne reaction products [76]. Nevertheless, such reactions have shown promising results to label cells, validating their significance to perform coupling reactions under physiological conditions.

### 3.1 Labeling of proteins

Strain-promoted [3 + 2] azide-alkyne cycloaddition reaction is a promising and noninvasive method for labeling settled and newly synthesized proteins in living systems. To enable the tagging of the proteome, non-canonical amino acids carrying reactive handles can be incorporated, allowing for subsequent detection of the labeled proteins [77,78]. One such example is covalent labeling with an alkyne-bearing fluorophore by copper-free click chemistry, enabling the visualization of newly synthesized proteins. Various reactive metabolic precursors having azido functional groups such as azido-proline analogs, methionine analogs such as azidohomoalanine (AHA), homopropargylglycine (HPG), and ethynylphenylalanine have been used for this approach, depending on the target proteins [77,79,80].

**Fig. 3:**
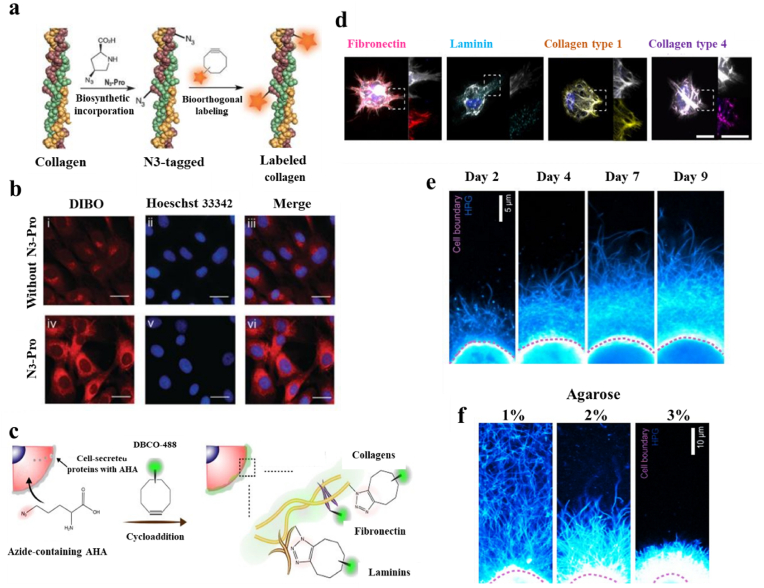
Protein visualization by metabolic labeling through bioorthogonal chemistry. **a)** Incorporation of N3-Pro (azide-proline) chemical reporter into collagen and labeling using a SPAAC bioorthogonal reaction with the fluorescent probe, **b)** Fluorescence labeling of proteins with DIBO in fetal ovine osteoblasts cultured in the absence (i–iii) or presence (iv–vi) of N3-Pro: (i) and (iv) staining with DIBO Alexa Fluor 555 (red); (ii) and (v) counter-staining with Hoechst 33,342 (blue); (iii) and (vi) overlay [85], **c)** Schematic of nascent extracellular protein labeling. The methionine analog, AHA, is added to the culture media and incorporated into nascent proteins. The biorthogonal Cu(I)-free strain-promoted cycloaddition between the azide and DBCO-modified fluorophore (DBCO-488) enables visualization of the proteins, **d)** Representative images (magnifications on the right) of nascent proteins and fibronectin, laminin α5 and collagen type I and type IV at 6 days [68], **e)** Images of chondrocytes cultured in 2% agarose and continuously labeled with HPG for up to 9 days and **f)** Images of HPG-labeled chondrocytes cultured in agarose of varying densities, demonstrating density of the cellular microenvironment impacts organization and distribution of nascent matrix [87]. (For interpretation of the references to colour in this figure legend, the reader is referred to the Web version of this article.)

Proline has been widely explored as a marker of protein biosynthesis in tissue engineering applications, especially for collagen which is the most abundant mammalian protein [26,27]. Azido-proline has been employed for residue-specific labeling of collagen model peptides by functionalization with triazolyl moieties [[81], [82], [83]]. Moreover, it has been proved that azido-proline is biocompatible, it does not disturb collagen triple helix formation, as well as it can be detected by a fluorescent probe [84]. Therefore, the azido-proline is one of the reporters that has been used for noninvasive imaging of collagen deposition through bioorthogonal chemistry. In an *in vitro* study by Amgarten et al. [85], ovine osteoblasts were selected as the model system to exploit the bioorthogonal imaging of collagen in live cells using dibenzooctyne (DIBO) as a fluorescent probe and exploiting a strain promoted azide–alkyne cycloaddition (SPAAC) bioorthogonal reaction (Fig. 3a). In this regard, ovine osteoblasts were cultured in 2D and two different concentrations of azido-proline added to the cultured fetal ovine osteoblasts. They demonstrated that the presence of azido-proline had no adverse effect on cell viability at either concentration, therefore the higher concentration was selected for bioorthogonal labeling. Additionally, the fluorescence microscopy images of azido-proline tagged collagen in fetal ovine osteoblasts after SPAAC reaction with the fluorescent probe DIBO showed a nearly 2-fold increase in fluorescence intensity compared to cells without azido-proline treatment (Fig. 3b). A relatively high background staining in the samples cultured in the absence of azido-proline can be detected, indicating unspecific DIBO labeling. Interestingly, confocal microscopy images on stained cells showed a dotted structure that likely corresponds to non-secreted pro-collagen, indicating that the cells take up the azide-modified proline. In another study, Bardsley et al. [86] used similar components, azido-proline and subsequent fluorescent alkyne (DIBO) to detect neocollagen produced by cells continuously *in vitro* without the need for the destruction of grafts. In this study, they seeded azido-proline treated cells onto 3D fluorescent poly (lactic-*co*-glycolic) acid (PLGA) scaffolds and placed constructs into a media containing Click-IT Alexa Fluor 488 DIBO Alkyne. Their findings demonstrated that the collagen produced by cells had been modified with azide-proline efficiently and the control group was shown to exhibit little background staining.

Besides, AHA has been employed in different research studies to label proteins containing methionine amino acids. For example, Loebel et al. [68] used AHA incorporation and a bioorthogonal SPAAC reaction with fluorophore-conjugated cyclooctyne (DBCO-488) to monitor the proteins deposition and their spatiotemporal presentation produced by bone marrow-derived human mesenchymal stromal cells (hMSCs) in a biodegradable HA-based hydrogel (Fig. 3c). Their results (Fig. 3d) illustrate that this bioorthogonal system could effectively label and monitor methionine-containing proteins including fibronectin, laminin α5 and collagen type I, and type IV produced by MSCs after 6 days of 3D culture in HA hydrogel. In another study, McLeod et al. [87] employed a similar bioorthogonal strategy using functional noncanonical amino acid tagging by AHA and HPG which are structurally and functionally similar to native methionine. To visualize proteins in both native cartilages and 3D-engineered constructs, the chondrocytes were cultured in agarose gels in the absence of native methionine and the continuous presence of either HPG or AHA. Following sample fixation, the incorporated HPG and AHA were identified by labeling alkyne or azide residues with fluorescent azide or alkyne tags, respectively. Their results demonstrated that HPG-tagged proteins could be visualized clearly and HPG incorporation did not alter the mechanical or functional properties of the newly formed matrix. Moreover, they assessed the time course of protein deposition of chondrocytes, and by increasing incubation time, the proteins progressively extended from the cell (Fig. 3e). Additionally, they proved that protein accumulation rate heavily depends on polymer integration, physical, and mechanical properties provided by the surrounding microenvironment (Fig. 3f). In a more recent study, Saleh et al. [88] used AHA labeling to identify and isolate labeled proteins from embryos that minimize background signals from unlabeled proteins. They injected pregnant C57BL/6 mice with AHA and then, AHA-labeled proteins from homogenized E12.5 and E15.5 embryos were conjugated with diazo biotin-alkyne. They could observe newly synthesized AHA-labeled proteins by 3 h with peak labeling around 6 h, and approximately 50% of all identified proteins were found only in AHA samples, compared with proteins present in both AHA and PBS samples. It showed the developed method in this work enables the identification and quantification of protein synthesis and turnover in different tissue fractions during development.

This bioorthogonal approach using proteins-segment analogs enables type-specific protein visualization*,* and it also allows the evaluation of different parameters such as biophysical, biochemical, and mechanical cues on protein formation and deposition. Thus, this strategy is a powerful tool for monitoring matrix accumulation in regenerative medicine approaches and diagnosing diseases where abnormal collagen production is the main problem, such as diabetic nephropathy, cirrhosis, or idiopathic pulmonary fibrosis.

### 3.2 Labeling of polysaccharides

The analysis of glycans has gained significant traction in the fields of biological research, clinical analysis, and pharmaceutical biotechnology production. This is due to the identification of distinct glycosylation patterns that are associated with a variety of health and disease states. As a result, glycan analysis has emerged as a valuable tool for investigating the molecular mechanisms underlying these conditions and developing novel therapeutic interventions [89]. There are several techniques available for glycan derivatization and labeling, including reductive amination, Michael addition, hydrazide labeling, and permethylation. Usually, these strategies followed by some aspects on purification of derivatized glycans. Additionally, various techniques have been explored for analyzing and quantifying tagged oligosaccharides, including chromatographic and electromigrative separation methods, as well as mass spectrometry (MS) [89].

**Fig. 4:**
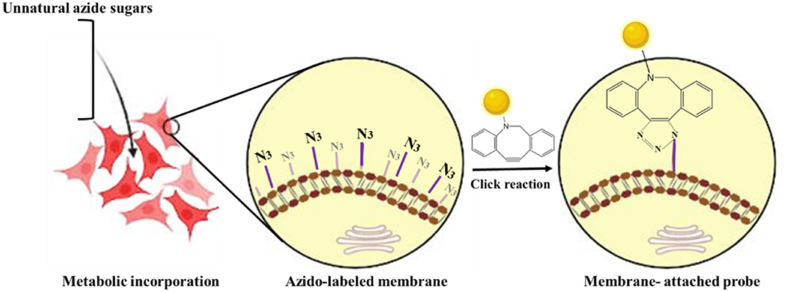
The schematic illustration of metabolic labeling using bioorthogonal chemistry. In the first step, the labeling agents such as unnatural azido sugars are fed to the cells that are then displayed on the cell surface after metabolizing the sugars. In the second step, the azido group site-selectively interact with a complementary bioorthogonal component such as DBCO bearing a probe which is coupled through a copper-free click reaction, allowing detection and imaging of the target cells.

Identification and quantification of glycans/polysaccharide formation can be also monitored by metabolic labeling with unnatural sugars followed by chemical reactions with imaging probes. Azide sugars such as *N-*azidoacetylmannosamine (ManNAz), *N*-azidoacetylgalactosamine (GalNAz), *N-*azidoacetylglucosamine (GlcNAz), and fucose‐containing glycans are abiotic azide-derivatives of naturally occurring monosaccharides which are mostly used for metabolic labeling of glycans [90]. As demonstrated in Fig. 4, the synthetic azide sugars are installed and azido groups are displayed on the cell surface. Consequently, the azido groups are covalently coupled to azide partners with fluorescent probes using a ‘click’ reaction [91,92]. This metabolic labeling strategy can be used for cell monitoring and selective imaging of cells and glycans for stem cell therapy, cancer-targeted therapy, immunomodulation, and regenerative medicine approaches [7,70,80,90,[92], [93], [94], [95], [96], [97], [98], [99]].

## 4 Application of ECM labeling

**Fig. 5:**
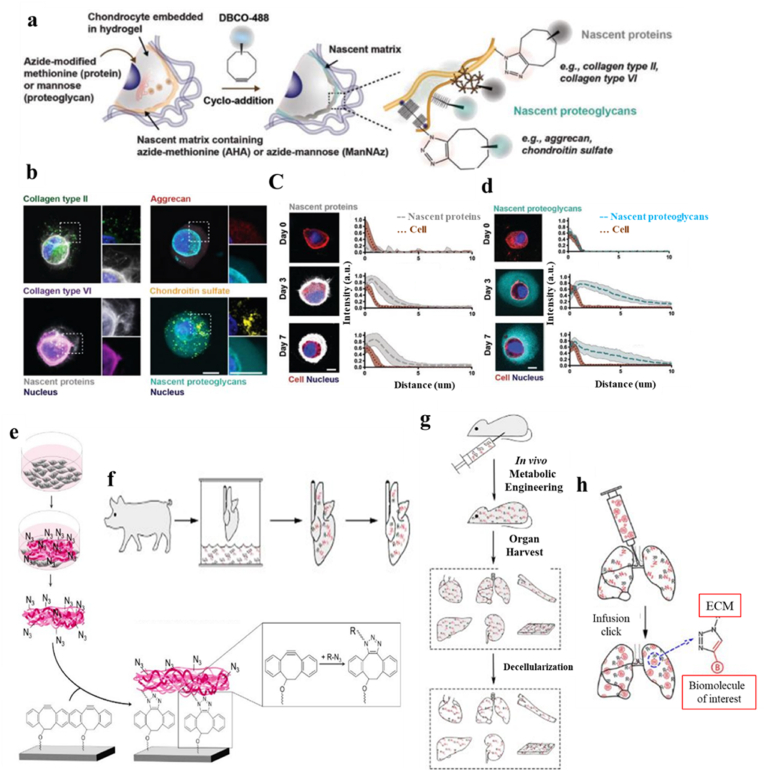
ECM labeling for its visualization and use as scaffold material. **a)** Schematic of nascent protein and proteoglycan metabolic labeling. The azide-modified methionine analog or mannose analog are added to the culture media and incorporated into nascent proteins (for example, collagen type II and collagen type VI) or proteoglycans (for example, aggrecan core protein and its associated chondroitin sulfate residues), respectively. The biorthogonal Cu(I)-free strain-promoted cycloaddition between the azide and DBCO-488 enables visualization of either the nascent proteins or proteoglycans, **b)** Representative images (magnifications on the right) of nascent proteins (white, co-stained with either collagen type II and VI) or proteoglycans (cyan, co-stained with aggrecan and chondroitin sulfate) deposited by bovine chondrocytes encapsulated in norbornene-modified HA hydrogels and cultured for 7 d in chondrogenic media (scale bars 10 μm), **c)** Representative images of accumulated nascent proteins of encapsulated chondrocytes cultured in chondrogenic media for up to 7 d (left, scale bars 10 μm) and radial intensity profiles of nascent proteins (gray). Lines represent median intensity profiles; shaded areas represent standard deviation, **d)** Representative images of accumulated nascent proteoglycans of encapsulated chondrocytes cultured in chondrogenic media for up to 7 d (left, scale bars 10 μm) and radial intensity profiles of nascent proteoglycans (cyan). Lines represent median intensity profiles, shaded areas represent standard deviation [69], **e)** Preparation of an azide-modified cell-derived ECM. Primary human fibroblasts were treated with an azide-modified sugar analog to metabolically incorporate azide groups into the cellular glycans. After 6 days of culture, the fibroblasts were lysed and the cytoplasm was washed off to purify the ECM. The chemical accessibility of the incorporated azide groups was demonstrated by immobilizing the ECM on an alkyne-functionalized silicon wafer [91], **f** and **g)** Diagram of metabolic labeling of porcine organ ECM *ex vivo* and *in vivo,* respectively, and **h)** Diagram of whole organ infusion click reaction allowing immobilization of alkyne-modified biomolecules onto azide-labeled acellular organ scaffolds (diagram showing the lung as an example) [99]. (For interpretation of the references to colour in this figure legend, the reader is referred to the Web version of this article.)

In the field of tissue engineering and regenerative medicine, ECM plays a critical role. ECM formation along with temporal and spatial positioning of proteins and polysaccharide-containing structures deposited by cells can indicate tissue regeneration status. Different methods such as immunohistochemistry, histology, and microscopy techniques have been used for ECM visualization [[100], [101], [102]]. However, the majority of them are destructive approaches and need a high number of samples for repetition. They do not allow live monitoring of ECM*,* and they cannot demonstrate the variation with time or the localization of the deposited matrix. Bioorthogonal chemistry enables noninvasive *in situ* monitoring of ECM*,* For instance, Loebel et al. [69] employed the metabolic labeling technique using bioorthogonal chemistry to probe the spatiotemporal accumulation of matrix deposited by chondrocytes. In this regard, chondrocytes were embedded into a crosslinked HA hydrogel and encapsulated fluorescent beads were placed into the hydrogel matrix to monitor the position of the hydrogel concerning the nascent matrix during culture. The constructs were cultured in media containing AHA or tetraacylated *N*-azidoacetyl-mannosamine to visualize proteins and proteoglycans, respectively, and then, harvested by first staining live cells using a fluorophore-conjugated cyclooctyne (DBCO-488) to fluorescently label AHA-containing proteins or *N*-azidoacetyl- mannosamine-tetraacylated -containing proteoglycans (Fig. 5a). Fig. 5b illustrates this labeling technique that enables visualization of all methionine- or mannosamine-containing matrix components produced by cells, supported by immunostaining to image collagen type II and type VI, or the proteoglycans such as aggrecan and associated chondroitin sulfate residues. Additionally, the results demonstrated that proteins and proteoglycans deposited with different spatial distribution after being secreted by chondrocytes within hydrogels, when cultured in chondrogenic media for 7 days, the thickness of this protein layer increased, indicative of those nascent proteins accumulated in the pericellular space. Labeling of proteoglycans also showed a similar deposition fashion around the cell body, however, the nascent proteoglycans extended further into the hydrogel at each time point (Fig. 5c and d). This approach is very useful for providing a broad investigation of ECM components synthesized and can prove beneficial for tissue regeneration and repair approaches. In summary, metabolic labeling using bioorthogonal chemistry enables monitoring ECM deposition noninvasively, and it also allows the location and position of ECM components to be detected at different time points which can provide new insights into the role of specific ECM components in tissue engineering and regenerative medicine approaches. In another work, Mao et al. [103] employed a bioorthogonal reaction-based strategy to investigate *in situ* specific and fast cellularization of tissue engineering scaffold. In this work, a DBCO-modified PCL-PEG (PCL-PEG-DBCO) polymer was synthesized through electrospinning. This polymeric scaffold was transplanted in mice *in vivo* and after 6 h macrophages that were labeled with tetraacylated *N*-azidoacetylmannosamine (Ac4ManNAz) were injected into the abdominal cavity of the same mice. They used this strategy to enable a controllable *in situ* cellularization of scaffolds to enhance their biological functions and tissue regeneration.

ECM-based biomaterial or cell-derived matrices have now emerged as a promising candidate to develop scaffold for tissue engineering applications [104]. ECM creates a 3D microenvironment that can be effectively modified to enhance mechanical stability. The matrix also supports the ability to stimulate cell adhesion and migration via exposed cell-binding sites thereby modulating cell processes and can act as a reservoir for growth factors [104,105]. However, ECM matrices lack specific functional groups and lack molecular triggers with the ability to guide cell function, regenerative properties, and post-implantation performance. The biological complexity of the cell-derived ECM challenges the incorporation of such functional groups without disturbing the integrity of the biomolecules within the ECM. Bioorthogonal chemistry has been employed for the functionalization and engineering of decellularized native ECM biomaterial for tissue engineering and regeneration approaches [64]. In this strategy, the ECM components are labeled by metabolic precursors such as unnatural sugars or non-canonical amino acids and then functionalized with the desired groups through click chemistry. In a research study, Ruff et al. [91] developed a cell-derived ECM with azide functionalities using bioorthogonal chemistry. Human primary fibroblasts isolated from human foreskin were cultured in the Dulbecco's Modified Eagle Medium (DMEM), supplemented with 10% fetal calf serum, penicillin/streptomycin, and Na-l-ascorbate. 1,3,4,6-tetra-*O*-acetyl-*N*-azidoacetylgalactosamine (50 μM) was added to the cell culture media and the flasks were incubated for 6 days (or 21 days for histological analysis) to produce an azide-modified ECM. Half of the culture medium was carefully removed every two to three days and replaced by fresh medium. The secreted azide-modified cells were fixed with formalin and later fluorescently labeled with an alkyne-modified fluorophore, employing a copper-catalyzed azide-alkyne cycloaddition reaction and detected using a confocal laser scanning microscope (Fig. 5e). In another study, Ren et al. [99] used a similar metabolic labeling method for chemoselective functionalization of native ECM to facilitate tissue regeneration and repair. The authors labeled the ECM using unnatural azide sugars both *in vivo* and *ex vivo* followed by a decellularization process (Fig. 5f and g). Briefly, for *in vivo* metabolic labeling, reagents were administered via intraperitoneal injection daily for 3 days (30 mg/day). One day after the last administration of the metabolic labeling reagents, organs were harvested from the animals and decellularized. For *ex vivo* labeling, freshly isolated porcine left lungs were cultured *ex vivo* in bioreactors for 24 h in the presence of GalNAz and decellularized. These experiments demonstrated that the azide-labeled acellular scaffolds can be immobilized of alkyne-modified biomolecules interest by click reaction (Fig. 5h).

Other research studies exist that utilized the bioorthogonal chemistry approach for labeling. Functionalization of ECM can be used to develop a bio-scaffold for different tissue regeneration approaches. Additionally, a similar strategy can be used to conjugate different molecules such as drugs and growth factors to the ECM scaffold for a more efficient regeneration and repair process. In Table 2 the major techniques and targets of these studies are illustrated.

**Table 2:** The summary of research studies utilized the bioorthogonal chemistry approach for labeling ECM molecules to functionalize ECM-based biomaterial or cell-derived matrices.

Authors	Cell/Tissue Type	Metabolic Precursors	Labeling Technique	Application/Target
Fruh et al. [106]	Human plasma	Azide-containing peptide (FKGGGK(N_3_))	Azide-labeled fibronectin was conjugated with DBCO-Alexa Fluor 488 through SPAAC	Site-specific incorporation of fluorophores probes into the ECM protein fibronectin by activated factor XIII
Ruff et al. [91]	Human fibroblasts	1,3,4,6-tetra-*O*-acetyl-*N*-azidoacetylgalactosamine	Azide-modified cell derived ECM was conjugated with DBCO	Conjugating the ECM with silicon wafer
Madl et al. [64]	Elastin-like proteins (ELPs)	Azide-bearing 4-phenyl-1,2,4-triazoline-3,5-dione (PTAD)	Azide-functionalized ELPs crosslinked with bicyclononyne functionalized ELPs via SPAAC	Azide-functionalize tyrosine residues within the proteins, selectively for bioorthogonal chemistry of protein hydrogels
Steen et al. [107]	Chondroitin sulfate	*N*-hydroxysuccinimide -PEG4-azide	Functionalized lyophilisome was generated by reaction between azido-conjugated lyophilisomes and labeled GD3G7 antibodies	Targeting the cancer ECM for an anti-cancer strategy to form a depot of chemotherapeutic-loaded lyophilisomes
Gutmann et al. [108]	NIH-3T3 fibroblasts	GalNAz and GlcNAz	Labeled-cell-derived matrix was featured for bioorthogonal conjugation chemistry by SPAAC or CuAAC	Site-specific decoration of matrix with bioactive molecules for tissue repair applications
Nellinger et al. [109]	Adipose-derived stem cells (ASCs)	1,3,4,6-tetra-*O*-acetyl-*N-*azidoacetylgalactosamine	Isolation of azide-modified ECM produced by ASCs and then detected through CuAAC using an alkyne-linked fluorophore	Labeled ECM of the ASCs can be used as a multifunctional biomaterial for bio-applications
Loebel et al. [69]	Chondrocytes	AHA and tetraacylated *N*-azidoacetyl- mannosamine	methionine- or mannose- containing matrix components were monitored by using DBCO-488	Probing the spatiotemporal accumulation of matrix deposited by chondrocytes encapsulated in the hydrogel
Keller et al. [110]	Human primary fibroblasts	1,3,4,6-tetra-*O*-acetyl-*N*-azidoacetylgalactosamine	Azide-labeled ECM was conjugated with alkyne-modified biotin molecules	Generation of azide-modified ECM as coating to create homogeneous, dense, and highly bioactive cell substrates
Jacome et al. [111]	Rat lung	Tetraacylated *N*-Azidoacetylgalactosamine	Azide-labeled ECM was conjugated with a desired alkyne-modified biomolecule, such as growth factors and glycosaminoglycans	Functionalization of native tissue-derived ECM by bioactive molecules for tissue engineering and wound repair

## 5 Pitfalls of ECM labeling and methods to overcome

Even with great advancements in biorthogonal labeling techniques in recent years, noted limitations still exist with many forms of biorthogonal labeling chemistry. First, bioorthogonal labeling chemistry involving Copper or other metal-catalyzed reactions has shown cytotoxic effects on cells [112]. Metal-free biorthogonal techniques have been developed to reduce the cytotoxic effects, but this remains a concern for newly adopted metabolic biorthogonal labeling chemistry [113]. All amino acids are incorporated in nearly every protein; therefore, identification of relative abundance of these proteins is of paramount importance to identify newly formed ECM. To a lesser extent this goes for the incorporation of sugar residues, but as glycosylation is an important posttranslational modification of proteins and extracellular glycoproteins are abundant, also here specificity is an issue that in most cases can only be solved by studying *in vitro* and *in vivo* processes characterized by the relative abundance of the protein of interest or using additional technologies to demonstrate relative specificity [65]. Biorthogonal labeling in itself may only be able to distinguish the newly formed ECM components, therefore it could be combined with other traditional methods such as immunofluorescent antibody labeling or liquid chromatography–mass spectrometry (LC-MS) or MS analysis to verify which type of proteins are secreted. Steps can also be taken to reduce errors in imaging the bioorthogonal labeled molecules by creating control samples with unlabeled species to provide a baseline normalization image, which needs to be performed on all culture environments and sample types included in one study. Additionally, multiple biorthogonal labeling techniques can be directly compared together by the simultaneous addition of different stable isotopes and methionine analogs. These methods can also be incorporated into sugar and metabolite labeling techniques to improve outcomes. Nonspecific labeling cannot be prevented entirely, but efforts have been made to improve upon the outcomes with the combinations of different labeling and quantification techniques [65].

Further limitations include poor labeling due to the insolubility of some proteins, especially ECM proteins. Click chemistry reactions like CuAAC used in metabolic labeling have been shown to have poor efficiency with targets having limited solubility [114]. To counter the poor solubility of some proteins for labeling, some researchers have had to resort to sonication to physically disrupt protein-to-protein interactions and improve labeling efficiency or add different molecules to interfere with normal protein secretion like high molar urea [88]. These methods, however, reduce the non-invasiveness of metabolic labeling techniques and will disrupt or destroy cellular components and physical features.

Other concerns of note with metabolic labeling of ECM components are physiological relevance and how the introduction of foreign biorthogonal chemicals into the system will perturb the results. Whenever a foreign chemical entity is introduced into a biological environment, that environment will undergo some form of change or disruption in response to that foreign species regardless of whether that chemical species is bioorthogonal. The cell is a crowded place with a high density of many types of molecules of different sizes including proteins, sugars, and lipids. When a foreign chemical used as a metabolic label is introduced into the biological system, that chemical can physically hinder other molecules' and proteins’ ability to bind or react by simply being in the way of native molecules as found under normal physiological conditions. Furthermore, a foreign chemical species may interact non-chemically with any number of other molecules and disrupt diffusion or change the pH of the system for example. The unnatural chemicals can directly compete with native metabolites and proteins. Furthermore, depending on the method of metabolic labeling used, the unnatural chemicals may be less efficiently incorporated or metabolized compared to the natural chemicals present and may lead to unwanted and problematic metabolites [115]. The size of the disruption from the introduction of bioorthogonal chemical species can cloud observations from studies involving bioorthogonal chemistry and might raise doubts about the actual physiological relevance of the system after labeling is introduced. Metabolic labeling of proteins and glycans in the ECM will leave a fingerprint in the system with a major shortcoming of how the unnatural molecules introduced will compete directly with native molecules, proteins, and other glycans in the system [115].

Challenges to overcome with biorthogonal chemistry ECM labeling techniques in the future include improving upon the efficiency and precise quantifiability of labeling techniques while also reducing any cytotoxic or cell-compromising and physiologically detrimental effects. Even if the ECM labeling chemistry remains biorthogonal, some effects may prove to be detrimental over longer term labeling due to bioaccumulation over time from the cell's inability to degrade byproducts of biorthogonal reactions. Additionally, distinguishing between or applying differential labeling between already existing ECM components and newly formed ECM pieces remains a challenge. Research teams have already begun addressing these issues by incorporating multiple forms of bioorthogonal labeling simultaneously such as protein labeling and glycosylation-enabled labeling [116]. With the combination of these challenges, researchers move forward in their techniques to improve upon our understanding of the ECM and its dynamics through development, regeneration, healing, and disease progression.

## 6 Conclusion and future direction

In conclusion, biorthogonal chemistry allows visualization and quantification of ECM component synthesis in living cells and tissues in real-time. By enabling quantification and visualization of the growing and living ECM, complex ECM dynamics can be more thoroughly investigated and understood. These techniques will help pave the way for better therapeutics that target ECM modalities and a wider understanding of how the ECM changes in various pathologies. Improving upon the challenges and limitations of bioorthogonal chemistry for labeling the ECM will further advance our understanding of the complexities involved in the ECM and related pathologies. Future directions in biorthogonal chemistry used in ECM labeling include combining different biorthogonal approaches simultaneously to deliver a more powerful understanding of the ECM with studies that combine glycomics, proteomics, and the interface between cells and biomaterials. Other possibilities include reducing the metabolic footprint of bioorthogonal chemistry introduced into living systems to improve upon the physiological relevance of the labeling method.

## Credit author statement

**Shima Tavakoli:** Conceptualization, writing original draft, visualization and editing, **Austin Evans:** Writing original draft, reviewing and editing, **Oommen P. Oommen:** Reviewing original draft and editing, **Laura Creemers:** Reviewing and editing, **Jharna Barman Nandi:** Reviewing and editing, **Jöns Hilborn:** Reviewing and editing, **Oommen P. Varghese:** Supervision, conceptualization, reviewing original draft and editing.

## Declaration of competing interest

The authors declare that they have no known competing financial interests or personal relationships that could have appeared to influence the work reported in this paper.

## Data Availability

No data was used for the research described in the article.

## References

[1] Anthony L. (2021).

[2] Bonnans C., Chou J., Werb Z. (2014). Remodelling the extracellular matrix in development and disease. Nat. Rev. Mol. Cell Biol..

[3] Kusindarta D.L., Wihadmadyatami H. (2018). Tissue Regen.

[4] Sándor G. (2013). Tissue engineering: propagating the wave of change. Ann. Maxillofac. Surg..

[5] Schiller J., Huster D. (2012). New methods to study the composition and structure of the extracellular matrix in natural and bioengineered tissues. Biomatter.

[6] Gold E.W. (1981). The quantitative spectrophotometric estimation of total sulfated glycosaminoglycan levels formation of soluble alcian blue complexes. BBA - Gen. Subj..

[7] Xie R., Hong S., Chen X. (2013). Cell-selective metabolic labeling of biomolecules with bioorthogonal functionalities. Curr. Opin. Chem. Biol..

[8] McKee T.J., Perlman G., Morris M., Komarova S.V. (2019). Extracellular matrix composition of connective tissues: a systematic review and meta-analysis. Sci. Rep..

[9] Yue B. (2014). Biology of the extracellular matrix: an overview. J. Glaucoma.

[10] Huang Y., Kyriakides T.R. (2020). The role of extracellular matrix in the pathophysiology of diabetic wounds. Matrix Biol. Plus..

[11] Rozario T., DeSimone D.W. (2010). The extracellular matrix in development and morphogenesis: a dynamic view. Dev. Biol..

[12] Theocharis A.D., Skandalis S.S., Gialeli C., Karamanos N.K. (2015). Extracellular matrix structure. Adv. Drug Deliv. Rev..

[13] Urbanczyk M., Layland S.L., Schenke-Layland K. (2020). The role of extracellular matrix in biomechanics and its impact on bioengineering of cells and 3D tissues. Matrix Biol..

[14] Hascall V.C., Majors A.K., De La Motte C.A., Evanko S.P., Wang A., Drazba J.A., Strong S.A., Wight T.N. (2004). Intracellular hyaluronan: a new frontier for inflammation?. Biochim. Biophys. Acta - Gen. Subj..

[15] Li Y., Li L., Brown T.J., Heldin P. (2007). Silencing of hyaluronan synthase 2 suppresses the malignant phenotype of invasive breast cancer cells. Int. J. Cancer.

[16] Papakonstantinou E., Roth M., Karakiulakis G. (2012). Hyaluronic acid: a key molecule in skin aging. Dermatoendocrinol..

[17] Iozzo R.V., Murdoch A.D. (1996). Proteoglycans of the extracellular environment: clues from the gene and protein side offer novel perspectives in molecular diversity and function. Faseb. J..

[18] Frantz C., Stewart K.M., Weaver V.M., Frantz C., Stewart K.M., Weaver V.M. (2010). The extracellular matrix at a glance. J. Cell Sci..

[19] Iozzo R.V., Schaefer L. (2015). Proteoglycan form and function: a comprehensive nomenclature of proteoglycans. Matrix Biol..

[20] Kresse H., Schnherr E. (2001). Proteoglycans of the extracellular matrix and growth control. J. Cell. Physiol..

[21] Lim C.C., Multhaupt H.A.B., Couchman J.R. (2015). Cell surface heparan sulfate proteoglycans control adhesion and invasion of breast carcinoma cells. Mol. Cancer.

[22] Theocharis A.D., Gialeli C., Bouris P., Giannopoulou E., Skandalis S.S., Aletras A.J., Iozzo R.V., Karamanos N.K. (2014). Cell-matrix interactions: focus on proteoglycan-proteinase interplay and pharmacological targeting in cancer. FEBS J..

[23] Pollock V. (2007). XPharm Compr. Pharmacol. Ref..

[24] Stephenson F.H. (2016). Calc. Mol. Biol. Biotechnol..

[25] Mouw J.K., Ou G., Weaver V.M. (2014). Extracellular matrix assembly: a multiscale deconstruction. Nat. Rev. Mol. Cell Biol..

[26] Gelse K., Pöschl E., Aigner T. (2003). Collagens - structure, function, and biosynthesis. Adv. Drug Deliv. Rev..

[27] Shoulders M.D., Raines R.T. (2009). Collagen structure and stability. Annu. Rev. Biochem..

[28] Wang K., Meng X., Guo Z. (2021). Elastin structure, synthesis, regulatory mechanism and relationship with cardiovascular diseases. Front. Cell Dev. Biol..

[29] Foster J.A. (2013). Encycl. Biol. Chem..

[30] Pankov R., Yamada K.M. (2002). Fibronectin at a glance. J. Cell Sci..

[31] Main A.L., Harvey T.S., Baron M., Boyd J., Campbell I.D. (1992). The three-dimensional structure of the tenth type III module of fibronectin: an insight into RGD-mediated interactions. Cell.

[32] Moretti F.A., Chauhan A.K., Iaconcig A., Porro F., Baralle F.E., Muro A.F. (2007). A major fraction of fibronectin present in the extracellular matrix of tissues is plasma-derived. J. Biol. Chem..

[33] Durbeej M. (2010). Laminins, Cell Tissue Res..

[34] Hallmann R., Horn N., Selg M., Wendler O., Pausch F., Sorokin L.M. (2005). Expression and function of laminins in the embryonic and mature vasculature. Physiol. Rev..

[35] Iorio V., Troughton L.D., Hamill K.J. (2015). Laminins: roles and utility in wound repair. Adv. Wound Care.

[36] Brösicke N., Faissner A. (2015). Role of tenascins in the ECM of gliomas. Cell Adhes. Migrat..

[37] Jones F.S., Jones P.L. (2000). The tenascin family of ECM glycoproteins: structure, function, and regulation during embryonic development and tissue remodeling. Dev. Dynam..

[38] Farhat W.A., Geutjes P.J. (2009). Biomater. Tissue Eng. Urol..

[39] Loureiro dos Santos L.A. (2017). Ref. Modul. Mater. Sci. Mater. Eng..

[40] Mecham R.P. (2008). Methods in elastic tissue biology: elastin isolation and purification. Methods.

[41] Maurer L.M., Ma W., Mosher D.F. (2016). Dynamic structure of plasma fibronectin. Crit. Rev. Biochem. Mol. Biol..

[42] Maeda M., Izuno Y., Kawasaki K., Kaneda Y., Mu Y., Tsutsumi Y., Nakagawa S., Mayumi T. (1998). Amino acids and peptides. XXXI. Preparation of analogs of the laminin- related peptide YIGSR and their inhibitory effect on experimental metastasis. Chem. Pharm. Bull..

[43] Weller A., Beck S., Ekblom P. (1991). Amino acid sequence of mouse tenascin and differential expression of two tenascin isoforms during embryogenesis. J. Cell Biol..

[44] Schvartz I., Seger D., Shaltiel S. (1999). Vitronectin, Int. J. Biochem. Cell Biol..

[45] Cho J.Y., Chak K., Andreone B.J., Wooley J.R., Kolodkin A.L. (2012). The extracellular matrix proteoglycan perlecan facilitates transmembrane semaphorin-mediated repulsive guidance. Genes Dev..

[46] Cole G.J., Halfter W. (1996). Agrin: an extracellular matrix heparan sulfate proteoglycan involved in cell interactions and synaptogenesis. Perspect. Dev. Neurobiol..

[47] Nitkin R.M., Smith M.A., Magill C., Fallon J.R., Yao Y.M., Wallace B.G., McMahan U.J. (1987). Identification of agrin, a synaptic organizing protein from Torpedo electric organ. J. Cell Biol..

[48] Roughley P.J., Mort J.S. (2014). The role of aggrecan in normal and osteoarthritic cartilage. J. Exp. Orthop..

[49] Wight T.N., Kang I., Merrilees M.J. (2014). Versican and the control of inflammation. Matrix Biol..

[50] Wight T.N., Kinsella M.G., Evanko S.P., Potter-Perigo S., Merrilees M.J. (2014). Versican and the regulation of cell phenotype in disease. Biochim. Biophys. Acta - Gen. Subj..

[51] Davies J.E., Tang X., Denning J.W., Archibald S.J., Davies S.J.A. (2004). Decorin suppresses neurocan, brevican, phosphacan and NG2 expression and promotes axon growth across adult rat spinal cord injuries. Eur. J. Neurosci..

[52] Rauch U., Feng K., Zhou X.H. (2001). Neurocan: a brain chondroitin sulfate proteoglycan. Cell. Mol. Life Sci..

[53] Frischknecht R., Seidenbecher C.I. (2012). Brevican: a key proteoglycan in the perisynaptic extracellular matrix of the brain. Int. J. Biochem. Cell Biol..

[54] Yamaguchi Y.U. (1996). Brevican: a major proteoglycan in adult brain. Perspect. Dev. Neurobiol..

[55] Hildebrand A., Romaris M., Rasmussen M., Heinegard D., Twardzik D.R., Border W.A., Ruoslahti E. (1994). Interaction of the small interstitial proteoglycans biglycan, decorin and fibromodulin with transforming growth factor β. Biochem. J..

[56] Zhang W., Ge Y., Cheng Q., Zhang Q., Fang L., Zheng J. (2018). Decorin is a pivotal effector in the extracellular matrix and tumour microenvironment. Oncotarget.

[57] Hedbom E., Heinegard D. (1989). Interaction of a 59-kDa connective tissue matrix protein with collagen I and II. J. Biol. Chem..

[58] Jian J., Zheng Z., Zhang K., Rackohn T.M., Hsu C., Levin A., Enjamuri D.R., Zhang X., Ting K., Soo C. (2013). Fibromodulin promoted in vitro and in vivo angiogenesis. Biochem. Biophys. Res. Commun..

[59] Mohammadzadeh N., Lunde I.G., Andenæs K., Strand M.E., Aronsen J.M., Skrbic B., Marstein H.S., Bandlien C., Nygård S., Gorham J., Sjaastad I., Chakravarti S., Christensen G., Engebretsen K.V.T., Tønnessen T. (2019). The extracellular matrix proteoglycan lumican improves survival and counteracts cardiac dilatation and failure in mice subjected to pressure overload. Sci. Rep..

[60] Igwe J.C., Gao Q., Kizivat T., Kao W.W., Kalajzic I. (2011). Keratocan is expressed by osteoblasts and can modulate osteogenic differentiation. Connect. Tissue Res..

[61] Hartmann U., Hülsmann H., Seul J., Röll S., Midani H., Breloy I., Hechler D., Müller R., Paulsson M. (2013). Testican-3: a brain-specific proteoglycan member of the BM-40/SPARC/osteonectin family. J. Neurochem..

[62] Kim H.P., Han S.W., Song S.H., Jeong E.G., Lee M.Y., Hwang D., Im S.A., Bang Y.J., Kim T.Y. (2014). Testican-1-mediated epithelial-mesenchymal transition signaling confers acquired resistance to lapatinib in HER2-positive gastric cancer. Oncogene.

[63] Zhang Z., Gupte M.J., Ma P.X. (2013). Biomaterials and stem cells for tissue engineering. Expet Opin. Biol. Ther..

[64] Madl C.M., Heilshorn S.C. (2018). Bioorthogonal strategies for engineering extracellular matrices. Adv. Funct. Mater..

[65] Loebel C., Saleh A.M., Jacobson K.R., Daniels R., Mauck R.L., Calve S., Burdick J.A. (2022). Metabolic labeling of secreted matrix to investigate cell–material interactions in tissue engineering and mechanobiology. Nat. Protoc..

[66] Sletten E.M., Bertozzi C.R. (2009). Bioorthogonal chemistry: fishing for selectivity in a sea of functionality. Angew. Chemie - Int. Ed..

[67] Bertozzi C.R. (2011). A decade of bioorthogonal chemistry. Acc. Chem. Res..

[68] Loebel C., Mauck R.L., Burdick J.A. (2019). Local nascent protein deposition and remodelling guide mesenchymal stromal cell mechanosensing and fate in three-dimensional hydrogels. Nat. Mater..

[69] Loebel C., Kwon M.Y., Wang C., Han L., Mauck R.L., Burdick J.A. (2020). Metabolic labeling to probe the spatiotemporal accumulation of matrix at the chondrocyte–hydrogel interface. Adv. Funct. Mater..

[70] Wang H., Mooney D.J. (2020). Metabolic glycan labelling for cancer-targeted therapy. Nat. Chem..

[71] Kolb H.C., Finn M.G., Sharpless K.B. (2001). Click chemistry: diverse chemical function from a few good reactions. Angew. Chemie Int. Ed..

[72] Zhang L., Chen X., Xue P., Sun H.H.Y., Williams I.D., Sharpless K.B., Fokin V.V., Jia G. (2005). Ruthenium-catalyzed cycloaddition of alkynes and organic azides. J. Am. Chem. Soc..

[73] Himo F., Lovell T., Hilgraf R., Rostovtsev V.V., Noodleman L., Sharpless K.B., Fokin V.V. (2005). Copper(I)-catalyzed synthesis of azoles. DFT study predicts unprecedented reactivity and intermediates. J. Am. Chem. Soc..

[74] Baskin J.M., Prescher J.A., Laughlin S.T., Agard N.J., Chang P.V., Miller I.A., Lo A., Codelli J.A., Bertozzi C.R. (2007). Copper-free click chemistry for dynamic in vivo imaging. Proc. Natl. Acad. Sci. U. S. A..

[75] Sletten E.M., Bertozzi C.R. (2008). A hydrophilic azacyclooctyne for cu-free click chemistry. Org. Lett..

[76] Zhang C., Dai P., Vinogradov A.A., Gates Z.P., Pentelute B.L. (2018). Site-selective cysteine–cyclooctyne conjugation. Angew. Chemie - Int. Ed..

[77] Dieck S.T., Muller A., Nehring A., Hinz F.I., Bartnik I., Schuman E.M., Dieterich D.C. (2012). Metabolic labeling with noncanonical amino acids and visualization by chemoselective fluorescent tagging. Curr. Protoc. Cell Biol..

[78] Johnson J.A., Lu Y.Y., Van Deventer J.A., Tirrell D.A. (2010). Residue-specific incorporation of non-canonical amino acids into proteins: recent developments and applications. Curr. Opin. Chem. Biol..

[79] Dieterich D.C., Link A.J., Graumann J., Tirrell D.A., Schuman E.M. (2006). Selective identification of newly synthesized proteins in mammalian cells using bioorthogonal noncanonical amino acid tagging (BONCAT). Proc. Natl. Acad. Sci. U. S. A..

[80] Grammel M., Dossa P.D., Taylor-Salmon E., Hang H.C. (2012). Cell-selective labeling of bacterial proteomes with an orthogonal phenylalanine amino acid reporter. Chem. Commun..

[81] Erdmann R.S., Wennemers H. (2012). Conformational stability of collagen triple helices functionalized in the Yaa position by click chemistry. Org. Biomol. Chem..

[82] Erdmann R.S., Wennemers H. (2010). Functionalizable collagen model peptides. J. Am. Chem. Soc..

[83] Erdmann R.S., Wennemers H. (2013). Conformational stability of triazolyl functionalized collagen triple helices. Bioorganic Med. Chem..

[84] Reddy G.K., Dhar S.C. (1992). Metabolic studies on connective tissue collagens in bone and tendon of adjuvant arthritic rat. Calcif. Tissue Int..

[85] Amgarten B., Rajan R., Martínez-Sáez N., Oliveira B.L., Albuquerque I.S., Brooks R.A., Reid D.G., Duer M.J., Bernardes G.J.L. (2015). Collagen labelling with an azide-proline chemical reporter in live cells. Chem. Commun..

[86] Bardsley K., Yang Y., El Haj A.J. (2017). Fluorescent labeling of collagen production by cells for noninvasive imaging of extracellular matrix deposition. Tissue Eng. C Methods.

[87] Mcleod C.M., Mauck R.L. (2016). High fidelity visualization of cell-to-cell variation and temporal dynamics in nascent extracellular matrix formation. Sci. Rep..

[88] Saleh A.M., Jacobson K.R., Kinzer-Ursem T.L., Calve S. (2019). Dynamics of non-canonical amino acid-labeled intra- and extracellular proteins in the developing mouse. Cell. Mol. Bioeng..

[89] Ruhaak L.R., Zauner G., Huhn C., Bruggink C., Deelder A.M., Wuhrer M. (2010). Glycan labeling strategies and their use in identification and quantification. Anal. Bioanal. Chem..

[90] Aich U., Yarema K.J. (2008). Glycoscience.

[91] Ruff S.M., Keller S., Wieland D.E., Wittmann V., Tovar G.E.M., Bach M., Kluger P.J. (2017). clickECM: development of a cell-derived extracellular matrix with azide functionalities. Acta Biomater..

[92] Zhang X., Zhang Y. (2013). Applications of azide-based bioorthogonal click chemistry in glycobiology. Molecules.

[93] Rocha B., Calamia V., Mateos J., Fernández-Puente P., Blanco F.J., Ruiz-Romero C. (2012). Metabolic labeling of human bone marrow mesenchymal stem cells for the quantitative analysis of their chondrogenic differentiation. J. Proteome Res..

[94] Wang H., Sobral M.C., Zhang D.K.Y., Cartwright A.N., Li A.W., Dellacherie M.O., Tringides C.M., Koshy S.T., Wucherpfennig K.W., Mooney D.J. (2020). Metabolic labeling and targeted modulation of dendritic cells. Nat. Mater..

[95] Agatemor C., Buettner M.J., Ariss R., Muthiah K., Saeui C.T., Yarema K.J. (2019). Exploiting metabolic glycoengineering to advance healthcare. Nat. Rev. Chem..

[96] Chang P.V., Dube D.H., Sletten E.M., Bertozzi C.R. (2010). A strategy for the selective imaging of glycans using caged metabolic precursors. J. Am. Chem. Soc..

[97] Han S.S., Kang S.W. (2020). Metabolic labeling of live stem cell for in vitro imaging and in vivo tracking. Methods Mol. Biol..

[98] Laughlin S.T., Bertozzi C.R. (2009). Imaging the glycome. Proc. Natl. Acad. Sci. U. S. A..

[99] Ren X., Evangelista-Leite D., Wu T., Rajab K.T., Moser P.T., Kitano K., Economopoulos K.P., Gorman D.E., Bloom J.P., Tan J.J., Gilpin S.E., Zhou H., Mathisen D.J., Ott H.C. (2018). Metabolic glycan labeling and chemoselective functionalization of native biomaterials. Biomaterials.

[100] Chuo S.T.Y., Chien J.C.Y., Lai C.P.K. (2018). Imaging extracellular vesicles: current and emerging methods. J. Biomed. Sci..

[101] Rickelt S., Hynes R.O. (2018). Antibodies and methods for immunohistochemistry of extracellular matrix proteins. Matrix Biol..

[102] Rupert D.L.M., Claudio V., Lässer C., Bally M. (2017). Methods for the physical characterization and quantification of extracellular vesicles in biological samples. Biochim. Biophys. Acta - Gen. Subj..

[103] Mao D., Zhang C., Kenry, Liu J., Wang X., Li B., Yan H., Hu F., Kong D., Wang Z., Liu B. (2020). Bio-orthogonal click reaction-enabled highly specific in situ cellularization of tissue engineering scaffolds. Biomaterials.

[104] S H., Sl L., S L.K., Hinderer S., Layland S.L., Schenke-Layland K. (2016).

[105] J. Taipale, tt Ajnd Jorma Keski, Growth factors in the extracellular matrix; Growth Factors extracell. matrix, n.d. 10.1096/fasebj.11.1.9034166.

[106] Früh S.M., Spycher P.R., Mitsi M., Burkhardt M.A., Vogel V., Schoen I. (2014). Functional modification of fibronectin by N-terminal FXIIIa-mediated transamidation. Chem. bio. chem..

[107] van der Steen S.C.H.A., Raavé R., Langerak S., van Houdt L., van Duijnhoven S.M.J., van Lith S.A.M., Massuger L.F.A.G., Daamen W.F., Leenders W.P., van Kuppevelt T.H. (2017). Targeting the extracellular matrix of ovarian cancer using functionalized, drug loaded lyophilisomes. Eur. J. Pharm. Biopharm..

[108] Gutmann M., Braun A., Seibel J., Lühmann T. (2018). Bioorthogonal modification of cell derived matrices by metabolic glycoengineering. ACS Biomater. Sci. Eng..

[109] Nellinger S., Keller S., Southan A., Wittmann V., Volz A.C., Kluger P.J. (2019). Generation of an azide-modified extracellular matrix by adipose-derived stem cells using metabolic glycoengineering. Curr. Dir. Biomed. Eng..

[110] Keller S., Keller S., Wörgötter K., Liedek A., Kluger P.J., Bach M., Tovar G.E.M., Tovar G.E.M., Southan A. (2020). Azide-functional extracellular matrix coatings as a bioactive platform for bioconjugation. ACS Appl. Mater. Interfaces.

[111] Reinoso Jacome E., Ling Z., Xing Y., Reinoso Jacome E., Fok S., Ren X. (2021). Bioorthogonal labeling and chemoselective functionalization of lung extracellular matrix. Bio-Protoc..

[112] Kennedy D.C., McKay C.S., Legault M.C.B., Danielson D.C., Blake J.A., Pegoraro A.F., Stolow A., Mester Z., Pezacki J.P. (2011). Cellular consequences of copper complexes used to catalyze bioorthogonal click reactions. J. Am. Chem. Soc..

[113] Wu D., Yang K., Zhang Z., Feng Y., Rao L., Chen X., Yu G. (2022). Metal-free bioorthogonal click chemistry in cancer theranostics. Chem. Soc. Rev..

[114] Besanceney-Webler C., Jiang H., Zheng T., Feng L., Soriano Del Amo D., Wang W., Klivansky L.M., Marlow F.L., Liu Y., Wu P. (2011). Increasing the efficacy of bioorthogonal click reactions for bioconjugation: a comparative study. Angew. Chemie - Int. Ed..

[115] Zaro B.W., Batt A.R., Chuh K.N., Navarro M.X., Pratt M.R. (2017). The small molecule 2-Azido-2-deoxy-glucose is a metabolic chemical reporter of O-GlcNAc modifications in mammalian cells, revealing an unexpected promiscuity of O-GlcNAc transferase. ACS Chem. Biol..

[116] Z L., Michael Hu X.R. (2022). Extracellular matrix dynamics: tracking in biological systems and their implications. J. Biol. Eng..

